# Thyroid hormone transport and metabolism are disturbed in the placental villi of miscarriage

**DOI:** 10.1186/s12958-023-01142-1

**Published:** 2023-11-15

**Authors:** Zhen Yu, Xinghao Feng, Zhongshan Lin, Xuan Li, Shiyue Su, Huiru Cheng, Yuanyuan Yang, Zhaolian Wei

**Affiliations:** 1 Department of Obstetrics and Gynecology, the First Affiliated Hospital of Anhui Medical University, No.218 Jixi Road, Hefei, 230022 Anhui China; 2 NHC Key Laboratory of Study on Abnormal Gametes and Reproductive Tract, Anhui Medical University, No.81 Meishan Road, Hefei, 230032 Anhui China; 3 Key Laboratory of Population Health Across Life Cycle (Anhui Medical University), Ministry of Education of the People’s Republic of China, No. 81 Meishan Road, Hefei, 230032 Anhui China; 4 Anhui Province Key Laboratory of Reproductive Health and Genetics, No.81 Meishan Road, Hefei, 230032 Anhui China; 5 Biopreservation and Artificial Organs, Anhui Provincial Engineering Research Center, Anhui Medical University, No.81 Meishan Road, Hefei, 230032 Anhui China

**Keywords:** Miscarriage, Angiogenesis, Thyroid hormone, Transthyretin, Deiodinase

## Abstract

**Background:**

It has been long known that thyroid hormone regulates placental villi development, which is associated with the occurrence of miscarriage. However, whether abnormal thyroid hormone metabolism and transport in placental villi are involved in miscarriage is still to be verified.

**Methods:**

Placental villi of elective terminations of pregnancies (ETPs) and miscarriage were collected. Proliferative activity and apoptosis of villi trophoblasts and angiogenesis were detected by TUNEL and immunochemistry. The expressions of thyroid hormone receptors (THRs), transthyretin (TTR), monocarboxylate transporter 8 (MCT8), organic anion transporting polypeptides 1A1 (OATP1A1), deiodinase 2 (Dio2) and Dio3 were examined by RT-PCR, Western blot, immunohistochemistry and immunofluorescence. JEG3 cell was treated with iopanoic acid (IOP), an inhibitor of Dio2 activity, the expressions of Dio2, placenta growth factor (PLGF) and sFlt1 were detected by RT-PCR and Western blot.

**Results:**

Cell proliferation was suppressed and apoptosis was increased in placental villi cytotrophoblasts of miscarriage. CD34^+^ vessel number and vascular endothelial growth factor (VEGF) protein abundance were decreased in miscarriage. In miscarriage group, the gene expression of *Dio2*, *Dio3*, *TTR* and *THRα*, but not *THRβ*, *MCT8* and *OATP1A1*, were downregulated. The protein abundances of TTR and THRα were downregulated in miscarriage group, but not THRβ. The protein abundance of Dio2 in miscarriage villi was decreased compared with that in ETP. In JEG3 cells, the gene expression of *PLGF* was decreased and the expression of *sFlt1* was increased in IOP treatment; The protein abundance of Dio2 was downregulated but the gene expression of *Dio2* was unaffected in IOP treatment.

**Conclusion:**

Thyroid hormone transport and metabolism in miscarriage were disturbed and may impaired angiogenesis of placental villi, which was associated with the occurrence of miscarriage.

## Introduction

Miscarriage remains one of the most common obstetrical complications with little known about its pathogenesis. Indeed, miscarriage occurs in 10-15% of all recognized pregnancies [1]. The known causes of abortion at present are genetic factors, endocrine disorders, uterine anatomical malformations and infection factors. However, approximately 50% of patients affected still cannot find a clear cause. Recently, the impact of thyroid hormones (THs) on pregnancy has received increasing attention [2, 3].

THs are essential for embryo implantation, placentation and fetal development. Thyroid hormones helped to maintain early pregnancy in threatened abortion [4]. In vitro, 3,5,3`-triiodothyronine (T3) was able to suppress apoptosis of early placental extravilli trophoblasts (EVTs) and regulate the invasive ability of EVTs in placentation by enhancing the expression of MMPs, fetal fibronectin and integrin αβIT3 [5–7]. Meanwhile, subclinical hypothyroidism is often associated with infertility and pregnancy loss [8].

Human hemochorial placenta forms a natural barrier where maternal-fetal exchanges take place. Both T3 and T4 were detectable in the fetal exocoelomic membrane and fetal tissues at 5–6 gestational weeks, before fetal thyroid hormone production, which began at 14–16 gestational weeks [9–11]. This phenomenon indicates that the cellular uptake of thyroid hormones by the villus is thus critical for both thyroid hormone action within this organ and for thyroid hormone transport to the fetus [12]. First, trophoblasts internalize thyroid hormones intracellularly and then thyroid hormones, mainly T3, bind to thyroid hormone receptors (THRs) in nuclei to finally exert their physiological effects. According to early studies, the expression of THRs in villi and decidua were down-regulated in spontaneous and recurrent abortions [13, 14]. However, the molecular mechanisms that take place in the feta-maternal interface associated with thyroid hormone transport in miscarriage are still largely unknown.

In early third trimester, placental monocarboxylate transporter 8 (MCT8) mRNA level was upregulated in intrauterine growth restriction (IUGR) compared with control group matched for gestational age [15]. In present study, we examined the difference in the expression of TH transport- and metabolism-related genes in placental villi between miscarriage and normal controls and evaluated angiogenesis of the placenta. Our result demonstrated that placental villus thyroid hormone metabolism and transport were disturbed in miscarriage, companied by impaired vessel development of the placenta.

## Materials and methods

### Placental villi collections

All patients received written consent before surgery, and all procedures and processes were approved by the Ethics Committee of the First Affiliated Hospital of Anhui Medical University (Hefei, China)(authorization number: S20200021).

Samples were collected in the outpatient gynecological operating room of the First Affiliated Hospital of Anhui Medical University. The enrollment criteria were as follows: miscarriage in women was defined as a couple having one or more consecutive pregnancy losses before 13 gestational weeks. Elective terminations of pregnancies (ETPs) were confirmed by ultrasound, intrauterine pregnancy with embryo and cardiac tube pulsation. On the basis of 2017 Guidelines of the American Thyroid Association for the diagnosis and management of thyroid disease during pregnancy and postpartum, all the group patients’ TSH levels in the peripheral blood were less than 4 μIU/ml. Patients diagnosed with hypothyroidism, hyperthyroidism or other endocrine disorders were excluded. The mean age of each group was as follows: miscarriage 27.1 ± 6.1 years and ETP 26.3 ± 7.5 years (*P* > 0.05). The ETP was matched with miscarriage in gestational day (53.6 ± 10.2 days VS 57.1 ± 8.5 days). In cases of miscarriage, surgery was performed within the first 24 h after diagnosis. All women selected in this research had no medical or family history. Chromosomal abnormalities in villi were detected immediately after the operation.

The villi were collected immediately after uterine aspiration and curettage. After the sorting of villi, the villi were divided into three parts and washed separately with saline several times to avoid the contamination of blood or others. One part of the tissues was used for immunohistochemistry and immunofluorescence after fixed in 4% paraformaldehyde. Approximately 30 mg of the samples was immediately fitted into epoxy pipes containing l ml RNA Later solution (Invitrogen, United States) for mRNA detection and the rest was placed in pipes and quick-freezed in liquid nitrogen for Western blot analysis or other experiments.

### Cell culture

JEG-3 cells were used to examine the effect of iopanoic acid (IOP), a specific activity inhibitor of Dio2, on the expression of PLGF and sFlt1. The cells were incubated in DMEM medium supplemented with 10% fetal bovine serum, 100U/ml of penicillin and 100 μg/ml streptomycin at 37℃ in 5% CO_2_-95% air. The cells then were treated with IOP (100 μM) for 24 h. The cells were then harvested for immunoblotting and RT-PCR to detect the expression of Dio2, PLGF and sFlt1.

### RT-qPCR

The RNA Prep Pure Tissue Kit (TIANGEN, China) was used according to the manufacturer’s protocol for total RNA extraction in placental villi. After added isopropanol for precipitating of RNA and washed the RNA in pre-cooled 75% ethanol for three times. NanoDrop 2000 was used to detected the concentration of RNA the agarose gel electrophoresis was performed to detect integrity of RNA with Gel Red staining. A Prime Script RT reagent kit (Takara, Japan) was used for reverse-transcribe 500 ng of total RNA into cDNA in 10 μl of the reaction system. The Lightcycler480 Real-time software (Roche, Japan) was used to perform real-time quantitative PCR in a 20 μl of reaction system. 18 S RNA was used as the reference. The sequences of the gene-specific primers used are listed in Table 1. Relative mRNA expression levels were determined by the 2^−ΔΔCt^ method.

**Table 1:** The primers of RT-PCR

Genes	Sequence (5’-3’)	Length	Species
18 S	Forward: CATTCGAACGTCTGCCCTATC Reverse: CCTGCTGCCTTCCTTGGA	137	Human
THRα1	Forward: AGGTCACCAGATGGAAAGCG Reverse: AGTGATAACCAGTTGCCTTGTC	136	Human
THRβ1	Forward: TGGGACAAACCGAAGCACTG Reverse: TGGCTCTTCCTATGTAGGCAG	86	Human
TTR	Forward: TGGGAGCCATTTGCCTCTG Reverse: AGCCGTGGTGGAATAGGAGTA	240	Human
Dio2	Forward: AGCAGACTACTGGTCTACTCAC Reverse: CACAGACTAATTTGCCTTGGGA	89	Human
Dio3	Forward: ATCCTCGACTACGCGCAAG Reverse: GGGATGATGTAGGGAGAGTCC	200	Human
OATP1A1	Forward: TAATGTGGGTGTACGTCCTAGT Reverse: GCTCCTGTTTCTACAAGCCCAA	142	Human
MCT8	Forward: CCACGCCTACGGTAGAGAC Reverse: CAGAGTTATGGATGCCGAAGATG	126	Human
PLGF	Forward: GAACGGCTCGTCAGAGGTG Reverse: ACAGTGCAGATTCTCATCGCC	187	Human
sFlt1	Forward: GTCGTGTAAGGAGTGGACCA Reverse: GCAGATTTCTCAGTCGCAGG	211	Human

### Immunohistochemistry

Placental villi sections (3 μm) were dewaxed and rehydrated, and then subjected to sodium citrate buffer for epitope retrieval. After blocked with 3% H_2_O_2_ at room temperature for endogenous peroxidase activity, goat serum was used to eliminate the nonspecific binding of the primary antibodies (the antibodies were listed in Table 2). Sections were then incubated with specific primary antibodies overnight. Reactivity was detected by using the MAXIN Ultrasensitive SP kit (MAXIN KIT-9710, China) and counterstained with hematoxylin. The slides were assessed by two observers blinded to grouping. Five nonconsecutive fields of each slide were taken by Zeiss system at a magnification of ×400.

The proliferative activity of trophoblasts was determined by immunohistochemistry staining for Ki67, and apoptosis was determined by TUNEL assay. For semiquantitative analysis, the rate of positive cells in villi of per field was counted. CD34 was used as the hallmark of vascular endothelium to evaluate placental angiogenesis, and the number of CD34^+^ blood vessels per field was counted. For TTR, VEGF, THRα and THRβ, the percentage of positive cells per field was counted.

**Table 2:** The information of antibodies

Antibodies	Company and Catalog number	Dilution
Ki67	Abcam (ab16667)	1:500
CD34	Abcam (ab81289)	1:5000
TTR	Abcam (ab75815)	1:1000
VEGF	Abcam (ab32152)	1:500
THRα	Abcam (ab53729)	1:400
THRβ	Abcam (ab5622)	1:400
β-tubulin	Abcam (ab6046)	1:2000
Dio2	Abcam (ab77779)	1:100 for IF 1:2000 for WB

### Immunofluorescence

The placental villi Sect (3 μm) were dewaxed with xylene and rehydrated in gradient ethanol (from 100 to 50%) and then immersed in 0.01% Triton-X 100 solution. After blocking with 1% BSA, the rehydrated-sections were treated with specific primary antibody incubation (Dio2, 1:500) at 4℃ for 12 h. After washing 3 times with PBS, the secondary antibody was added and incubated for 1.5 h. Nuclear DNA was stained with DAPI. All the images were visualized on a full-field digital slice scanner (Pannoramic MIDI, Hungary).

### Western blot

Cytoplasmic protein of placenta villi and cells was isolated and the detailed method has been reported in previous study [16]. Steps were as follows: Placental villi and cells were homogenized in lysis buffer and homogenates were then centrifuged at 15,000 g for 15 min. Supernatants from each sample were added to a gel loading buffer and boiled for 10 min. After electrophoresis in 12.5% SDS-polyacrylamide gel, The gel was transferred electrophoretically onto a polyvinylidene fluoride membrane. The PVDF membranes were blocked by skim milk for 2 h at room temperature, and then incubated with primary antibodies (as listed in Table 2) for 3 h. After washes in DPBS containing 0.05% Tween-20 four times for 10 min each and PBS for 10 min once, the membranes were incubated with for 1.5 h at room temperature. The membranes were then washed four times in DPBS containing 0.05% Tween-20 for 10 min each and PBS for 10 min once, followed by signal development using an enhanced chemiluminescence detection kit.

### Statistical analysis

All statistical analysis was performed with SPSS 23.0 software. All quantified data were presented as means ± standard deviation (SD) after determination if samples were normally distributed using 1-sample K-S test. The independent two sample t-test was performed to compare the difference if samples were normally distributed. If samples were non-normal distribution, data were present as quartile range, the difference was compared by Mann-Whitney U test. All experiments were performed at least three times. Difference was considered to be significant for *P <* 0.05.

## Results

### Demographic characteristics

No family or medical history was permitted in women selected in present study. Those with thyroid diseases were all excluded by detecting serum TSH in outpatients. TSH levels in the two groups were similar (Table 3), and all were within the normal ranges. The average gestational day of the miscarriage group was 57.1 ± 8.5 days, and that of the ETP group was 53.6 ± 10.2 days (*P* > 0.05).

**Table 3:** Basic information and sera THs level of patients

	ETP (n = 20)	Miscarriage (n = 25)
GD (days)	53.6 ± 10.2	57.1 ± 8.5
Age (years)	26.3 ± 6.2	27.1 ± 7.5
TSH (μIU/mL)	1.63 ± 0.74	1.89 ± 0.82
T3 (nmol/L)	1.73 ± 0.28	1.93 ± 0.34
T4 ( nmol/L)	111.85 ± 16.27	124.01 ± 21.89

### Cell proliferation and apoptosis of trophoblasts in placental villi

**Fig. 1:**
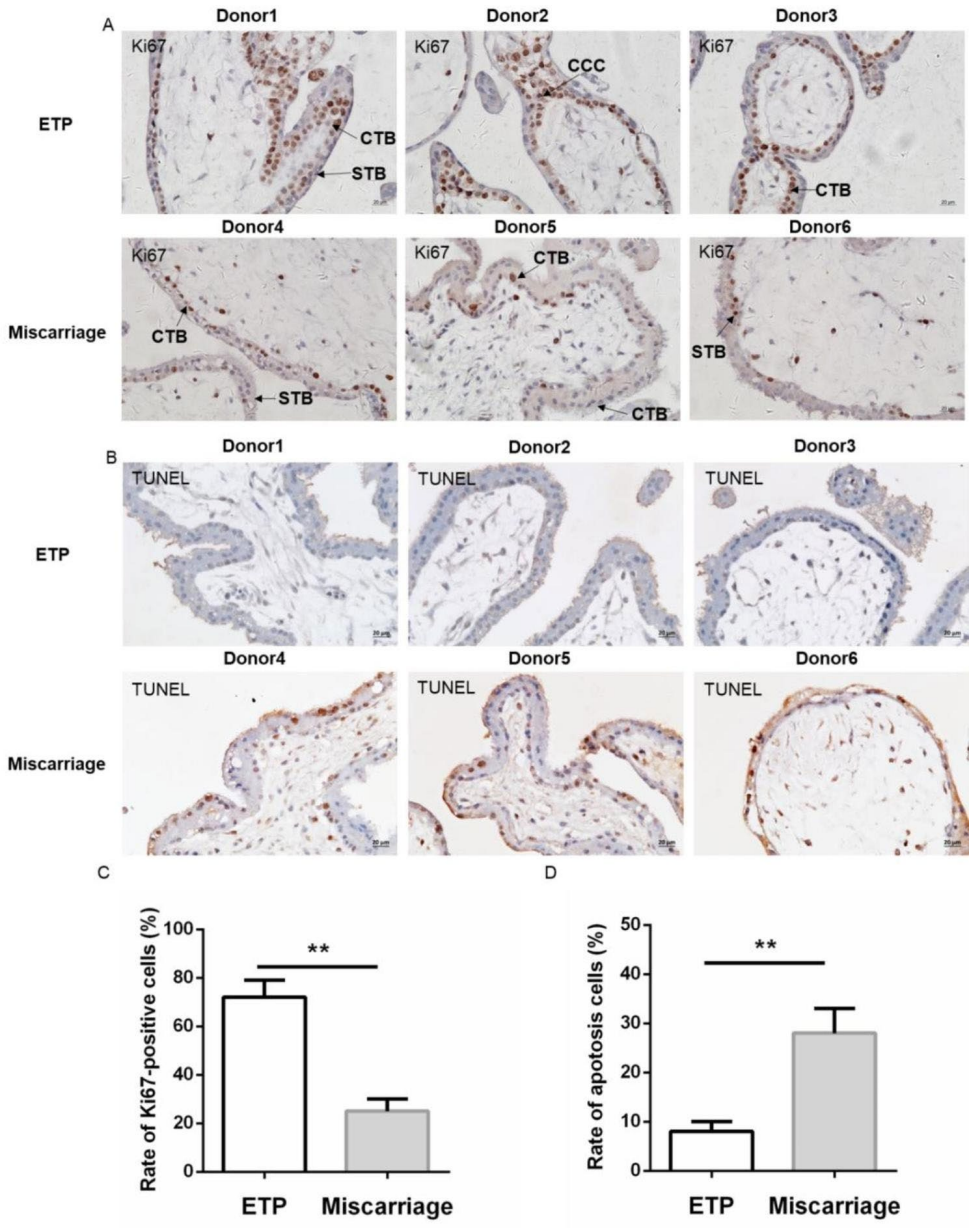
Cell proliferation was inhibited and apoptosis was increased in placental villi of miscarriage. (**A**) Immunohistochemistry for Ki67 was performed to evaluate cell proliferation in villi. Original magnification: 400×. (**B**) Placental villi sections were stained with TUNEL assay to assess cell apoptosis. Original magnification: 400×. (**C**) Rate of Ki67-positive cells. (**D**) Rate of apoptosis cell. Data were presented as the means ± SD (n = 20). **: *P* < 0.01. ETP: elective terminations of pregnancy. STB: syncytiotrophoblast. CTB: cytotrophoblast. CCC: cytotrophoblast cell columns

The Ki67 was used to reflect cell proliferation in villi. As shown in Fig. 1A, Ki67-positive cells were identified in villi cytotrophoblast (CTB) and cytotrophoblast cell columns (CCCs), but not in syncytiotrophoblasts (STB). Meanwhile, the rate of Ki67-positive nuclei was memorably reduced in villi of miscarriage as compared with the ETP group (Fig. 1C), indicating that cell proliferation in placental villi of miscarriage was suppressed.

The TUNEL assay was used to label fragmented nuclear DNA in nuclei to reflect the apoptosis of trophoblasts in placental villi. Similarly, as shown in Fig. 1B, the positive cells in villi were mainly VCTs and fewer positive nuclei were observed in the EPT group. Accordingly, the rate of positive cells was markedly increased in the miscarriage group (Fig. 1D), indicating that cell apoptosis in the placental villi of miscarriage was increased.

### Angiogenesis in placental villi

**Fig. 2:**
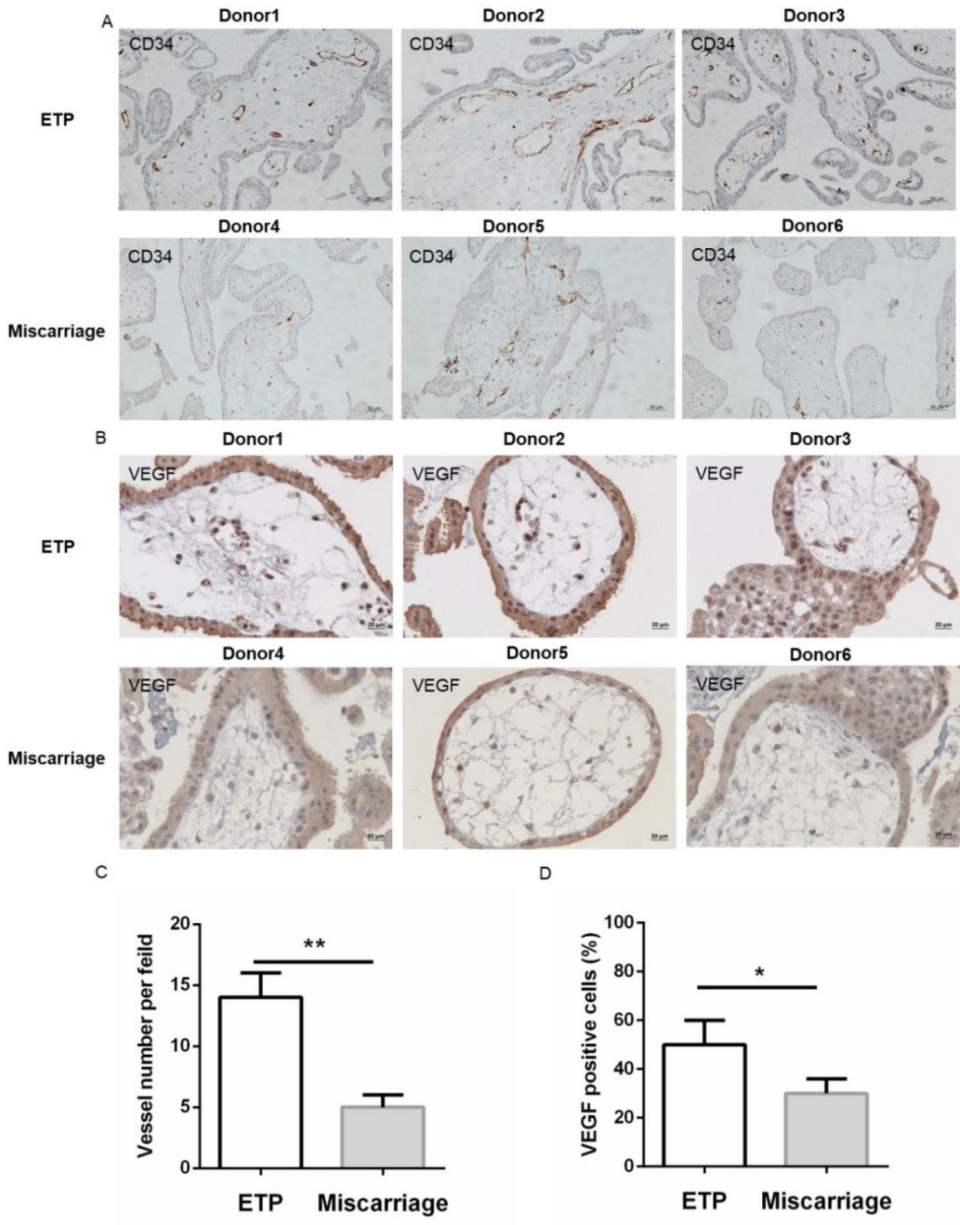
Angiogenesis was disturbed and the expression of VEGF was decreased in placental villi of miscarriage. (**A**) Immunohistochemistry staining for CD34. Original magnification: 200×. The arrows showed the vessel. (**B**) Immunohistochemistry staining for VEGF. Original magnification: 400×. (**C**) Quantitative analysis of CD34 positive vessel. (**D**) Rate of VEGF-positive cells in placental villi. Data were shown as the mean ± SD (n = 20).*: *P* < 0.05; **: *P* < 0.01. ETP: elective terminations of pregnancy

Angiogenesis is crucial for placentation and fetal development and disturbed angiogenesis in the placenta villus is associated with adverse birth outcomes, including pregnancy loss. As shown in Fig. 2A and **C**, the number of CD34^+^ vessels per field in placental villi was decreased in the miscarriage group than in the ETP group.

Additionally, we detected the protein expression of VEGF in placental villi by immunohistochemistry. As shown in Fig. 2B and **D**, the protein abundance of VEGF in placental villi of miscarriage was markedly decreased as compared to controls, demonstrating that angiogenesis in placental villi of miscarriage was affected.

### The expression of TTR in placenta villi

**Fig. 3:**
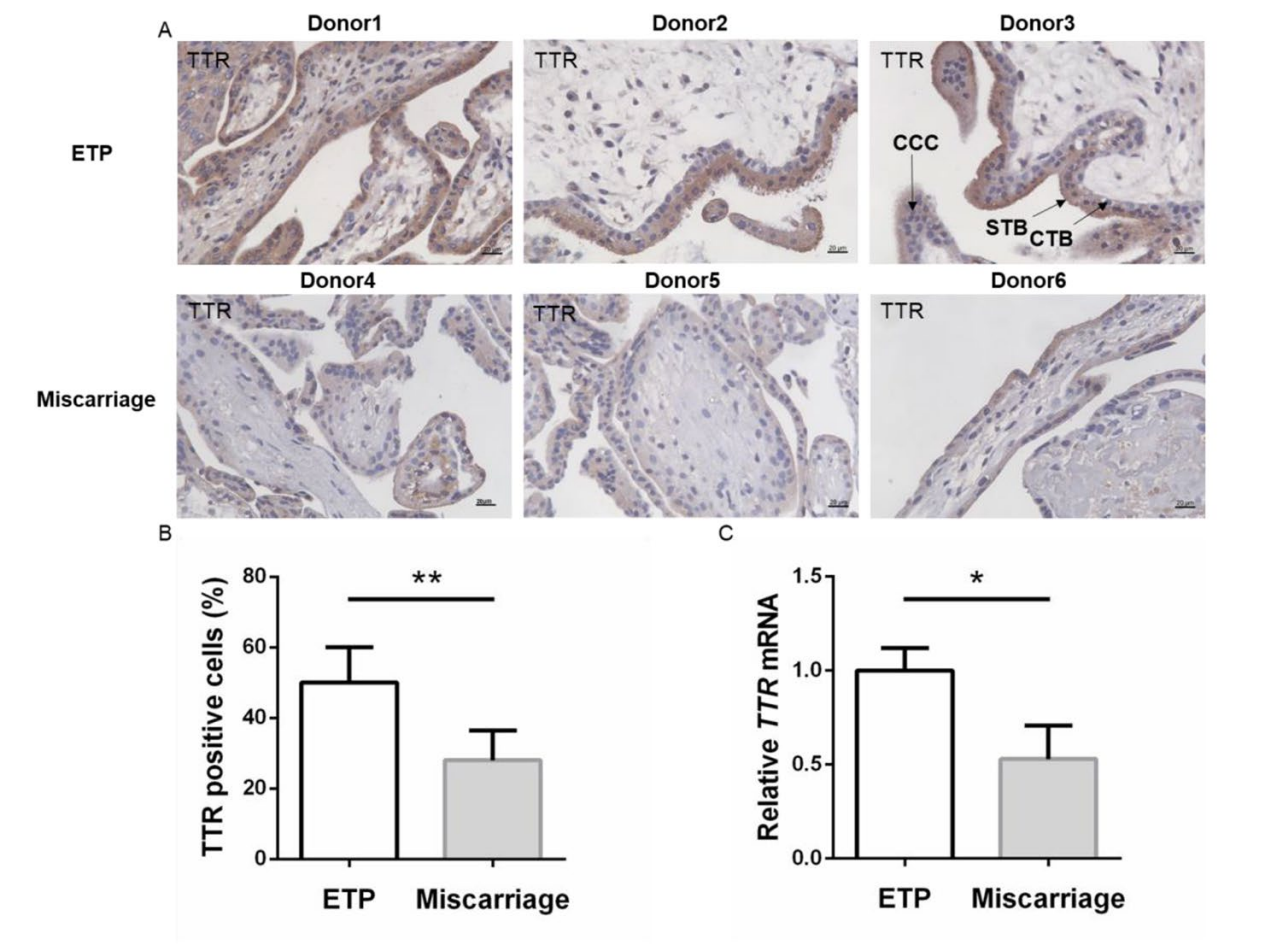
The expression of TTR was downregulated in placental villi of miscarriage. (**A**) Placental villi sections were stained with TTR. Original magnification: 400×. (**B**) Rate of TTR positive cells in placental villi; (**C**) The mRNA level of *TTR*. Data were shown as the mean ± SD (n = 20). *: *P* < 0.05. ETP: elective terminations of pregnancy. STB: syncytiotrophoblast. CTB: cytotrophoblast. CCC: cytotrophoblast cell columns

Transthyretin (TTR) is important in transporting thyroid hormones from maternal circulation to fetus through placenta. In early pregnancy, the need for thyroid hormones for placental and fetal development absolutely depends on transplacental passage. The expression of TTR in placenta villi was detected in this study. As shown in Fig. 3A, the immunohistochemistry results indicated that TTR was expressed in both cytotrophoblasts and syncytiotrophoblasts. The protein abundance of TTR in villi of miscarriage was significantly downregulated compared to ETP. Accordingly, mRNA level of TTR in the placenta villi of miscarriage patients was decreased compared with that in controls (Fig. 3D). This result implied that the transport of thyroid hormones through placenta in the miscarriage was disturbed.

### Thyroid hormone transport and metabolism in human placental villi

**Fig. 4:**
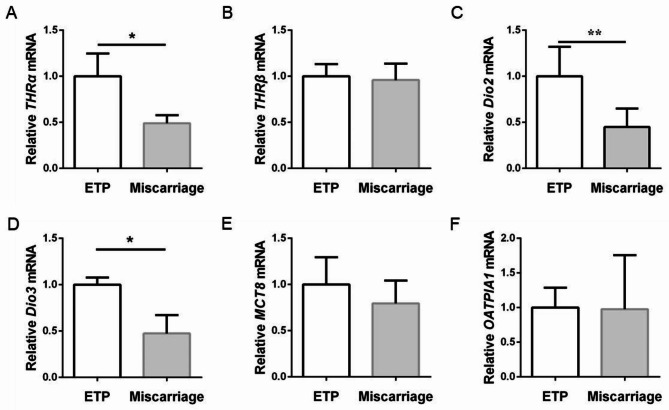
The expression of thyroid hormone receptors, deiodinases and thyroid hormone transporters in placental villi was detected. The mRNA level was measured using RT-PCR. (**A**) *THRα*; (**B**) *THRβ*; (**C**) *Dio2*; (**D**) *Dio3*; (**E**) *OATP1A1*; (**F**) *MCT8*. All data were expressed as the mean ± SD (n = 20). *: *P* < 0.05; **: *P* < 0.01. ETP: elective terminations of pregnancy

**Fig. 5:**
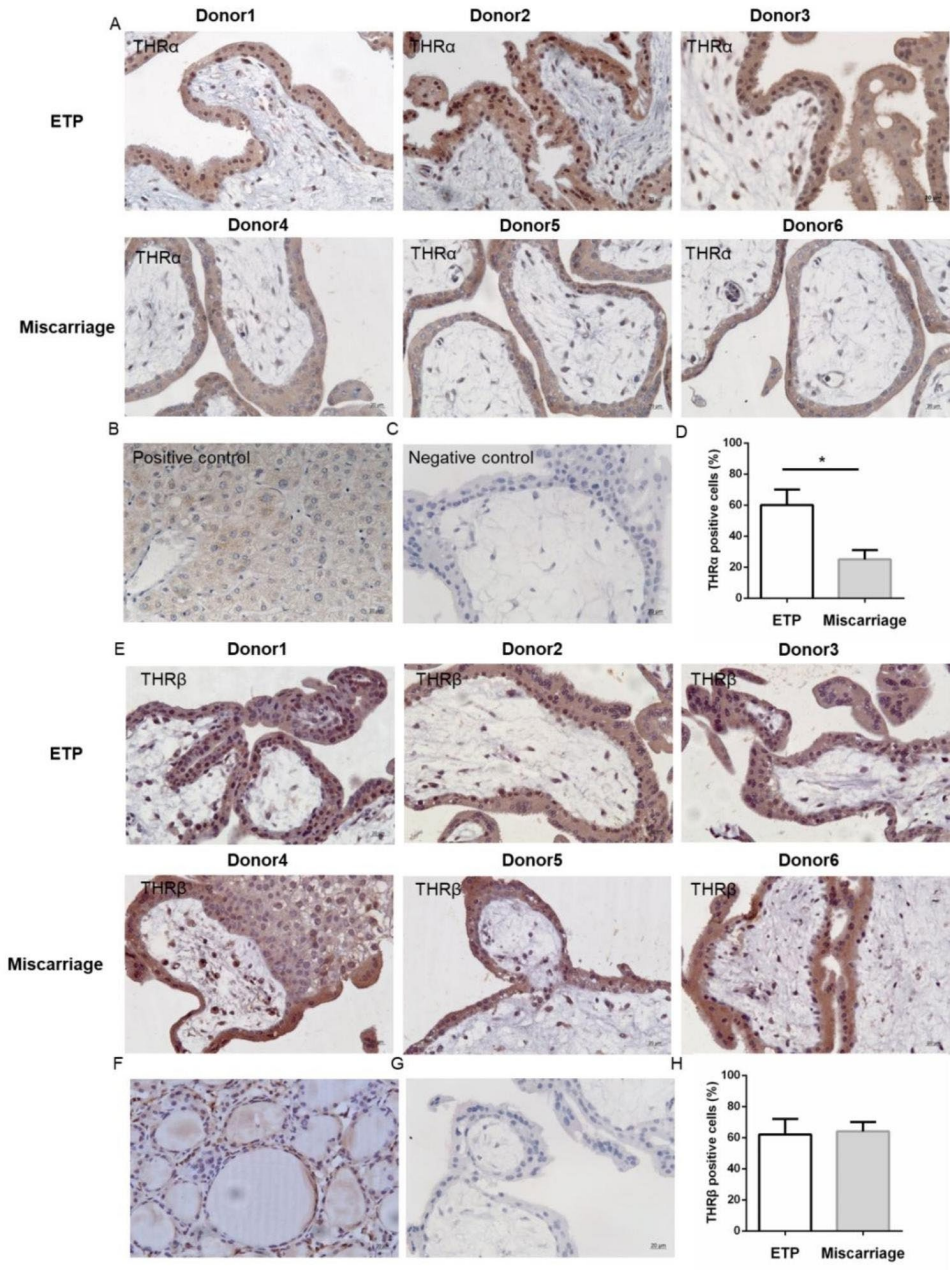
The expression of THRα was downregulated in placental villi of miscarriage but not THRβ. (**A**) Immunohistochemistry staining for THRα. Sections were counterstained with hematoxylin. Original magnification: 400×. (**B**) Sections from thyroid tumors were used as positive control. (**C**) PBS was used instead of primary antibody in the negative control. (**D**) Rate of THRα-positive cells in placental villi. (**E**) Immunohistochemistry staining for THRβ. Sections were counterstained with hematoxylin. Original magnification: 400×. (**F**) Sections from thyroid tumors were used as positive control. (**G**) PBS was used instead of primary antibody in the negative control. (**H**) Rate of THRβ-positive cells in placental villi. Data were shown as the mean ± SD (n = 20). *: *P* < 0.05. ETP: elective terminations of pregnancy

Placental development is also thyroid hormone dependent and it has been proven that the placenta expresses thyroid hormone transporters and deiodinases. We detected the expression of thyroid hormone transporters (*MCT8* and *OATP1A1*), deiodinases (*Dio2* and *Dio3*) and thyroid hormone receptors (*THRα* and *THRβ*) by RT-PCR. As shown in Fig. 4C **and D**, the expressions of *Dio2* and *Dio3* were significantly down-regulated in villi of the miscarriage group compared to the ETP group. No significant difference in villi thyroid hormone transporters (*MCT8* and *OATP1A1*) was detected. The expression of *THRα*, but not *THRβ*, was significantly decreased in the placental villi of miscarriage patients. Accordingly, the protein abundance of THRα in villi was also significantly downregulated in the miscarriage group, but not the protein abundance of THRβ (Fig. 5).

**Fig. 6:**
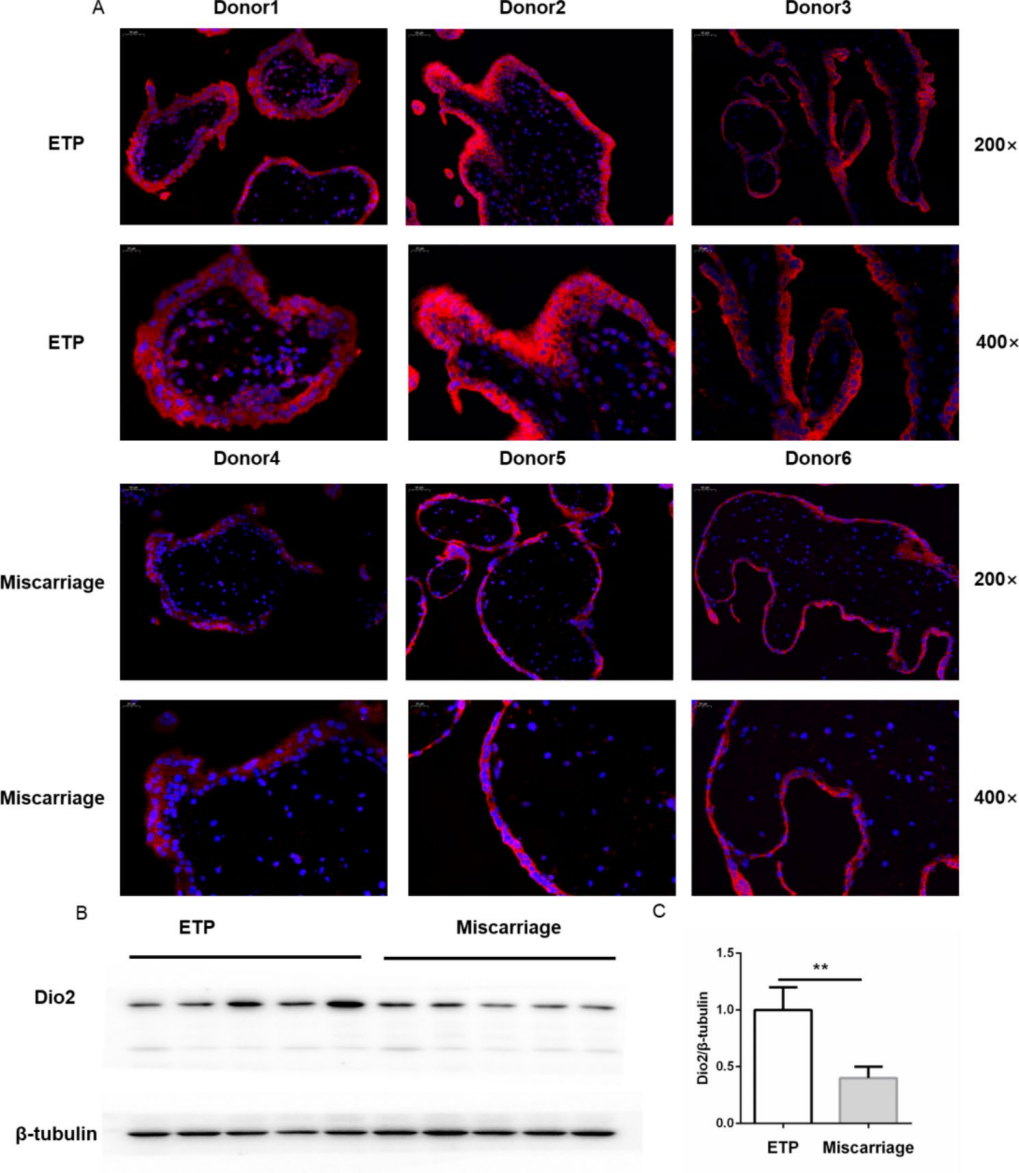
The expression of deiodinase 2 was decreased in placental villi of miscarriage. (**A**) Immunofluorescence detection of Dio2 in placental villi. DNA was counterstained with DAPI. Original magnification: 200× and 400×. (**B**) Immunobloting analysis of Dio2 in placental villi. All experiments were performed for at least three times. (**C**) Quantitative analyses of densitometry. Data were shown as the mean ± SD (n = 20). *: *P* < 0.05. ETP: elective terminations of pregnancy

Meanwhile, we detected the protein abundance of Dio2 in placental villi. As shown in Fig. 6A, the fluorescence intensity of Dio2 in placental villi of miscarriage was lower than that in ETP. Additionally, the western blot results demonstrated that the expression of Dio2 was downregulated in the placental villi of miscarriage patients (Fig. 6B and **C**). These results indicated that placental thyroid hormone metabolism and transplancental passage were affected in the miscarriage group.

### IOP disturbed the expressions of angiogenesis factors in JEG3 cells

**Fig. 7:**
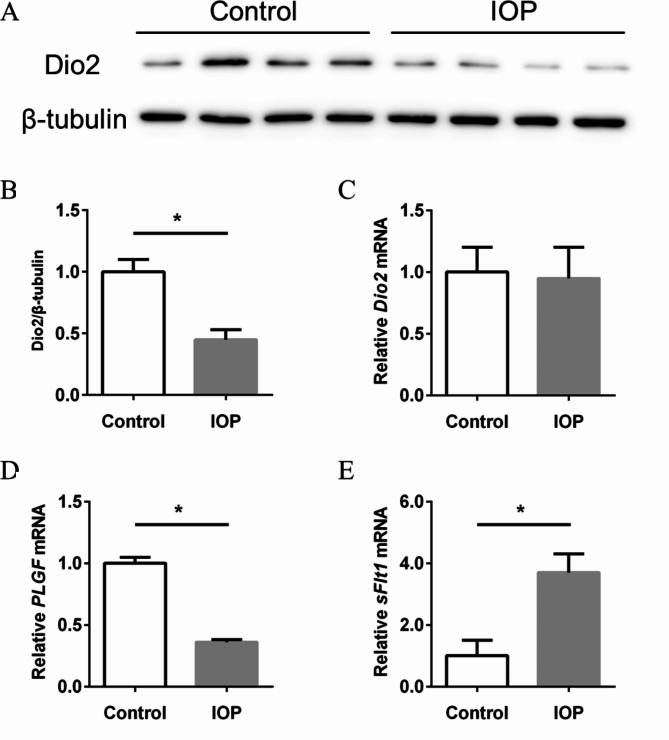
IOP treatment disturbed the expression of PLGF and sFlt1 in JEG3 cell. (**A**) Immunobloting analysis of Dio2. JEG3 cells were treated with IOP (100 μM) for 24 h, the protein abundance of Dio2 was detected by Western blot. (**B**) Quantitative analyses of densitometry. (**C**-**E**) the gene expression of Dio2, PLGF and sFlt1 was examined by RT-PCR. (**C**) *Dio2*; (**D**) *PLGF*; (**E**) *sFlt1*. Data were shown as the mean ± SD (n = 20). *: *P* < 0.05

IOP is the specific activity inhibitor of Dio2. The JEG3 cells were treated with IOP (100 μM) for 24 h to explore the effects on expression of PLGF and sFlt1. We found that the protein expression of Dio2 was decreased after IOP treatment but the mRNA level of *Dio2* was unchanged (Fig. 7A-C). As shown in Fig. 7D and E, the gene expression of *PLGF* was decreased and the gene expression of *sFlt1* was increased after the treatment of IOP. These result indicated that disturbed cellular thyroid hormone receptor signaling would impair the angiogenesis of placental villi, as downregulation of Dio2 can perturb the expression of angiogenesis factors.

## Discussion

A birth cohort study reported that thyroid dysfunction before gestational 20 weeks would lead to fetal loss and abnormal development [17]. Interestingly, a Chinese population based RCT (randomized clinical trial) undergoing IVF-ET whose thyroid function was in the normal range and positive for anti-thioperoxide antibodies, found that treatment with levothyroxine did not rescue the miscarriage [18]. Here, we hypothesized that the thyroid hormone transport- and metabolism-associated system in placenta villi was altered in the process of miscarriage. In particular, we found that abnormal maternal-fetal thyroid hormone transport caused by low expression of transthyretin in trophoblasts was involved in miscarriage.

The balance between proliferation and apoptotic process was crucial for placentation and there are many kinds of factors involved in this important process for normal fetal development. Thyroid hormones (THs) are important for the maintenance of normal pregnancy [19], minor fluctuations can result in many obstetric complications. The results from animal model showed hypothyroidism suppressed the proliferation rate the luteal cells and endothelial cells, and the effects of hypo-and hyperthyroidism on proliferation, apoptosis and expression of angiogenic factors among the pregnant rats were distinct [20]. In vitro, T3 regulated the proliferation and apoptosis of fetal-derived trophoblasts, and promoted the invasive ability of extravillous trophoblasts (EVTs) [5, 21]. In the present study, the proliferation and apoptosis of trophoblasts in placenta villi were examined. Our results showed that Ki67-positive nuclei were significantly reduced in cytotrophoblasts of miscarriage placental villi, and numerous apoptotic cells in miscarriage were counted. Cytotrophoblasts differentiate into different kinds of trophoblasts, such syncytiotrophoblasts and EVTs, that can form a biological placenta to support embryo growth. This demonstrated that the occurrence of abortion is at least partially related to the imbalance in proliferation and apoptosis of trophoblasts.

The placenta villus regulates maternal-fetal transport of thyroid hormones through the complex TH signaling pathway, which consists of the interaction of cellular membrane transporters, like MCT8 and OATPIA1, deiodinating enzymes and binding proteins, such as deiodinase 2 and 3 (Dio2 and Dio3) and TTR. However, how TH signaling regulates placentation and handles the occurrence of miscarriage is still unknown. Several epidemiological investigations has shown that maternal thyroxine deficiency is associated with complications of pregnancy, especially early pregnancy loss [17, 22–24]. The acquisition of fetal THs in early pregnancy was completely dependent on the transport of THs occurring in the maternal-fetal interface. However, much of the T4 experienced Dio3 was translated to biologically inactive reverse T3 (rT3). Protected by TTR, parts of T4 were converted to biologically active T3 by Dio2. Active T3 then exerts effects by binding to TH receptors, and promotes fetal development. It has been proved that THRα1 mutant mice exhibited pygmyism with increased mortality, reduced fertility and a smaller litter size [25]. Another animal study found that mice deficient in Dio3 showed impaired clearance of TH and resulted in the death of mice. Interestingly, MCT8 deficiency eliminated this consequence [26]. The expression of thyroid hormone receptors (THRα and THRβ) in placenta villi and decidual tissues in spontaneous abortion and recurrent spontaneous abortion was downregulated [13]. In vitro, the placenta could secrete TTR into the maternal circulation, which was beneficial for binding to their corresponding receptor [12, 27, 28]. Proteomic analysis based on one human study identified 13 proteins including TTR, that were downregulated in the placental villi of early pregnancy loss [29]. Surprisingly, we found that the expression of THRβ was not different between the miscarriages and ETP group. Our results showed lower expression of TTR and THRα in placenta villi in miscarriage cases. In addition, Dio2 and Dio3 presented lower expression at the mRNA level in the villi of miscarriage cases. These results showed that the dysregulation of complex interactions of membrane transporters, deiodinating enzymes and binding proteins, which would eventually lead to alteration in thyroid hormone metabolism and transportation, was at least partially involved in miscarriage.

Angiogenesis was the most essential factor affecting fetal and placental development and placental angiogenesis was dependent upon various growth factors (including VEGF and its receptors) and a number of signaling pathways. A previous study reported that the expression of hypoxia-inducible factor 1-alpha (HIF-1α), VEGF and microvessel density was decreased in missed abortion [30]. In an animal model, we found that DEHP exposure during pregnancy impaired placental angiogenesis leading to IUGR and the expression of THRs and VEGF was lower in the DEHP-treated group [16]. In another model, DEHP exposure disrupted the formation of labyrinth vascularization at gestational day 9 with reduced numbers of embryo implantation sites [31]. HIF-1α is the target gene of THRs and HIF-1α regulates the expression of VEGF. Cellular thyroid hormone receptor signaling is regulated by thyroid transporter, intracellular T3 concentration and the expression of thyroid hormone receptors. In this study, we treated JEG3 cells with IOP and found that decreased Dio2 expression could lead to decreased PLGF expression and increased sFlt1 expression. PLGF participated in the regulation of placental angiogenesis, sFlt1 is a soluble fragment of FLT1, which antagonizes angiogenesis. These results suggest that interference with intracellular thyroid hormone receptor signaling can lead to altered expression of angiogenic factors. Thus, altered thyroid hormone receptor signaling in placental villi is associated with reduced angiogenesis. Indeed, we found that villi angiogenesis was disrupted and the expression of THRα and VEGF was downregulated in miscarriage tissues.

There were still some limitations in our study. First, we clarified the expression of Dio2, and did not take Dio3 into consideration. Dio3 is an important regulator in controlling the transplancental adsorption of thyroid hormone from maternal circulation. In the first trimester, the expression of Dio3 was 100–300 times higher than that of Dio2. Second, we did not examine the cause of abnormal thyroid hormone transport and metabolism in placental villi. In our previous study, we found that maternal exposure to DEHP (a common used phthalate) during pregnancy would disturb the expression of THRs and VEGF. An epidemiologic study proved that phthalate exposure increased the risk of pregnancy loss [32]. As exposure to phthalate is common during pregnancy, we should pay more attention to the effects of environmental endocrine disruptors on the miscarriage and other adverse birth outcomes.

## Conclusions

Taken together, our results suggested that thyroid hormone transport and metabolism in placental villi of miscarriage were disturbed and would impaired angiogenesis of placenta, which may be associated with the occurrence of miscarriage.

## Data Availability

The datasets used and/or analyzed during the current study are available from the corresponding author on reasonable request.

## Abbreviations

CCC: Cytotrophoblast cell columns
CTB: Cytotrophoblast
Dio2/3: Deiodinase 2 and 3
ETP: Elective terminations of pregnancy
EVT: Extravillous trophoblast
IUGR: Intrauterine growth restriction
MCT8: Monocarboxylate transporter 8
OATP1A1: Organic anion transporting polypeptide 1A1
STB: Syncytiotrophoblast
TH: Thyroid hormone
THR: Thyroid hormone receptor
TTR: transthyretin
